# Umbilical cord-derived mesenchymal stem cells reversed the suppressive deficiency of T regulatory cells from peripheral blood of patients with multiple sclerosis in a co-culture – a preliminary study

**DOI:** 10.18632/oncotarget.12345

**Published:** 2016-09-29

**Authors:** Hongna Yang, Jinhua Sun, Feng Wang, Yan Li, Jianzhong Bi, Tingyu Qu

**Affiliations:** ^1^ Department of Critical-Care Medicine, Qilu Hospital of Shandong University, Shandong University, Jinan, Shandong, China; ^2^ Department of Psychiatry, College of Medicine, University of Illinois at Chicago, Chicago, IL, USA; ^3^ Department of Neurology, The Second Hospital of Shandong University, Shandong University, Jinan, Shandong, China; ^4^ R & D, Cell and Tissue Bank of Shandong Province, Jinan, Shandong, China

**Keywords:** umbilical cord mesenchymal stem cells, stem cell priming, multiple sclerosis, T regulatory cells, immunomodulation, Immunology and Microbiology Section, Immune response, Immunity

## Abstract

The immunoregulatory function of T regulatory cells (Tregs) is impaired in multiple sclerosis (MS). Recent studies have shown that umbilical cord-derived mesenchymal stem cells (UC-MSCs) exert regulatory effect on the functions of immune cells. Thus, we investigated whether UC-MSCs could improve the impaired function of Tregs from MS patients. Co-cultures of UC-MSCs with PBMCs of MS patients were performed for 3 days. Flow cytometry was used to determine the frequency of Tregs. A cell proliferation assay was used to evaluate the suppressive capacity of Tregs. ELISA was conducted for cytokine analysis in the co-cultures. Our results showed that UC-MSCs significantly increased the frequency of CD4^+^CD25^+^CD127^low/−^ Tregs in resting CD4^+^ T cells (p<0.01) from MS, accompanied by the significantly augmented production of cytokine prostaglandin E2, transforming growth factor (−β1, and interleukin-10, along with a reduced interferon-γ production in these co-cultures (p<0.05 - 0.01). More importantly, UC-MSC-primed Tregs of MS patients significantly inhibited the proliferation of PHA-stimulated autologous and allogeneic CD4^+^CD25^−^ T effector cells (Teffs) from MS patients and healthy individuals compared to non-UC-MSC-primed (naïve) Tregs from the same MS patients (p<0.01). Furthermore, no remarkable differences in suppressing the proliferation of PHA-stimulated CD4^+^CD25^−^ Teffs was observed in UC-MSC-primed Tregs from MS patients and naïve Tregs from healthy subjects. The impaired suppressive function of Tregs from MS can be completely reversed in a co-culture by UC-MSC modulation. This report is the first to demonstrate that functional defects of Tregs in MS can be repaired *in vitro* using a simple UC-MSC priming approach.

## INTRODUCTION

Multiple sclerosis (MS) is a chronic inflammatory, demyelinating, and neurodegenerative disorder of the central nervous system (CNS) [1], which affects approximately 400,000 people in the United States and 2.5 million worldwide [2]. Although oral treatment and parenteral administration exist, these treatments are expensive and also have toxic effects. The etiology of MS is currently not clear, evidence now reveals that the autoimmune response mediated by the invasion of auto reactive myelin-specific CD4^+^ T cells is a key pathological feature of MS. Auto-reactive T cells present in MS patients' blood penetrate the blood-brain barrier (BBB) and become active in the central nervous system (CNS) [1]. More importantly, the reduced number and/or function of T regulatory cells (Tregs) is responsible for the activation of auto-effective T cells in the peripheral and CNS [3-5].

Tregs play an important role in establishing and maintaining immunologic self-tolerance and immune homeostasis by various mechanisms. These include direct inhibition of auto-reactive T cell activation by secreting immunosuppressive mediators through cell-to-cell contact and indirectly via inhibition of the stimulatory capacity of antigen-presenting cells (APCs) [6]. Many studies report that the adoptive transfer of autologous or allogenic Tregs reversed or inhibited autoimmune disease development and controlled the allo-immune response to organ and cell transplantation by cell-to-cell contact, secretion of anti-inflammatory cytokines, and modulation of APCs [7-9]. Transplantation of chloroquine-elicited and allogenic Tregs reduced the severity of EAE (experimental autoimmune encephalomyelitis) in an animal model of MS [10]. Pre-clinical and clinical studies have shown that autologous Tregs may have potential as a novel therapy in the treatment of autoimmune diseases. However, studies have confirmed that Tregs, directly from the patients with autoimmune diseases including MS, cannot efficiently inhibit the proliferation of auto-effective T cells because of their impaired suppressive function [11, 12]. Therefore, developing clinically applicable protocols that reverse or repair the impaired suppressive function of these Tregs would be necessary before autologous Tregs could be used as real therapeutic cells.

Mesenchymal stem cells (MSCs) as immunomodulatory cells have been receiving attention because MSCs are able to induce FoxP3+ expression in CD4 T cells *in vitro* and increase the proportion of Tregs *in vivo* [13-15]. Some groups have reported that the immunosuppressive function of CD4^+^CD25^+^ Tregs or CD8^+^CD28^−^ Tregs from PBMCs of healthy donors are enhanced *in vitro* by co-culture with allogeneic MSCs from bone marrow (BM) [16, 17]. MSCs from human umbilical cords (UC-MSCs) are bioequivalent to MSCs from bone marrow. In fact, UC-MSCs are genome stable and have lower immunogenicity and higher expansion ability compared with those from BM and other adult tissues [18, 19]. UC-MSCs are also able to promote the production of Tregs *in vitro* [20] and to increase peripheral Treg*in vivo* [21]. In our previous studies, we demonstrated that UC-MSCs significantly increase the frequency of CD4^+^CD25^high^CD45RA^+^ Tregs and the production of anti-inflammatory cytokines in co-cultures with PBMCs from healthy subjects [22]. In the current study, we further investigated the immunomodulatory effects of UC-MSCs on the frequency and immunosuppressive function of Tregs from the peripheral blood of MS patients by co-cultures of UC-MSCs and PBMCs of MS patients.

## RESULTS

### UC-MSCs significantly increased the frequency of Tregs among resting CD4^+^ T cells derived from PBMCs of MS patients

**Figure 1:**
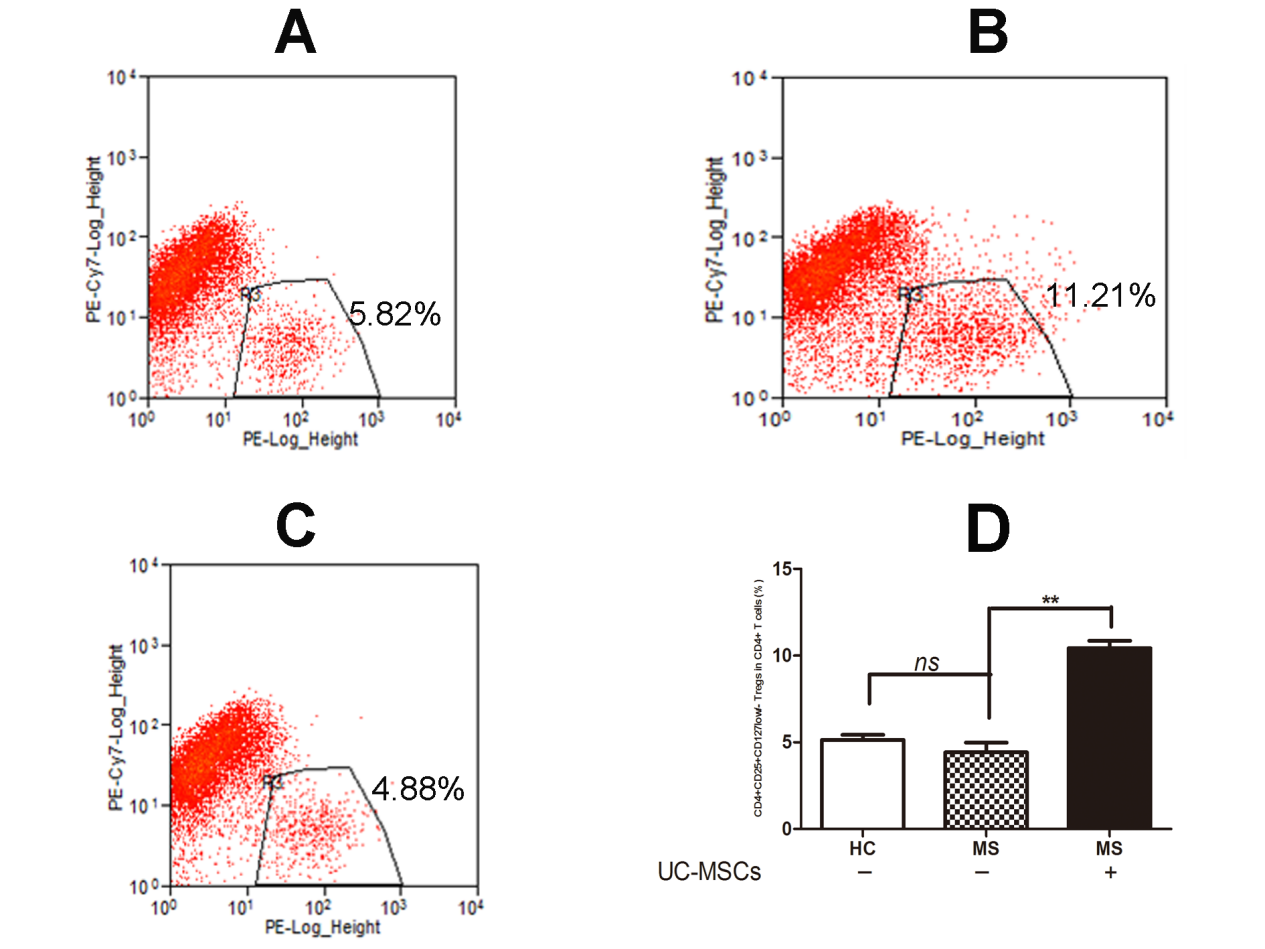
UC-MSCs significantly improved the frequency of Tregs among resting CD4^**+**^ T cells from MS **A**, **B**, & **C**. Representative dot plot for CD25^+^CD127^low/−^ Tregs among resting CD4+ T cells in cultured PBMCs of healthy donors (A), MS patients (B) following 3-day co-culture without UC-MSCs and (C) with UC-MSCs (UC-MSCs:PBMCs=1:5). **D**. Bar graph showing that the frequency of CD4^+^CD25^+^CD127^low/−^ Tregs among resting CD4+ T cells in PBMCs of MS was significantly increased following a 3-day co-culture with UC-MSCs compared to controls without UC-MSCs. No significant differences in the frequency of CD4^+^CD25^+^CD127^low/−^ Tregs were detected in naïve healthy donors and naïve MS patients. The data were expressed as mean ± SD of four experiments. **p<0.01. MS: multiple sclerosis, HC: healthy controls.

We examined the frequency of CD4+CD25+CD127low/− Tregs in PBMCs from MS patients and healthy controls. No significant difference was detected for the frequency of CD4+CD25+CD127low/− Tregs in CD4+ T cell sub-populations from MS compared to healthy controls. However, the frequency of CD4+CD25+CD127low/− Tregs in CD4^+^ T cells of MS patients was significantly increased in co-cultures with UC-MSCs compared to those without UC-MSCs (Figure 1D, **p<0.01). Further examination showed that UCMSC co-cultures significantly increased the frequency of CD4+CD25+CD127low/− Tregs not only in naïve PBMCs but also in PHA-activated PBMCs from MS (data not shown).

### UCMSC co-culture reversed the impaired suppressive function of Tregs from MS

**Figure 2:**
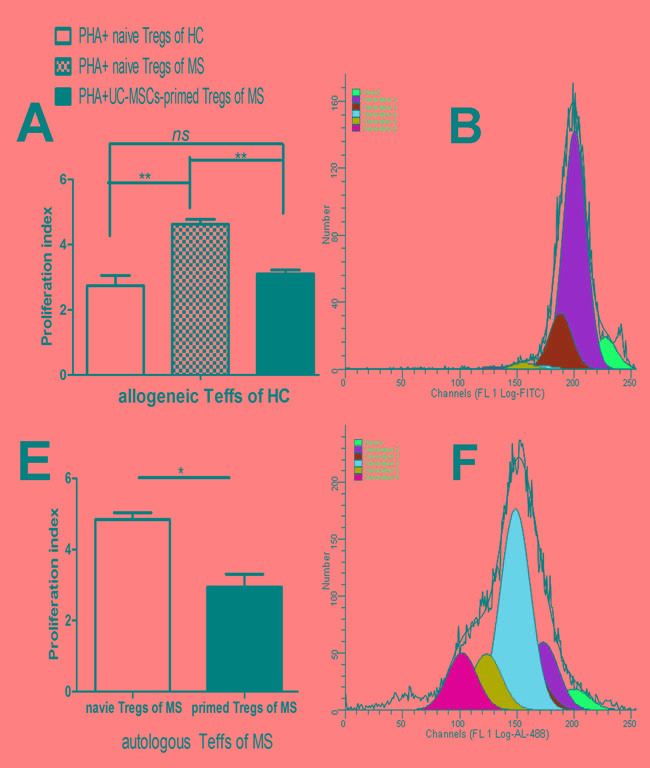
UC-MSCs reversed the impaired suppressive function of Tregs from MS **A**. Bar graph showing that Tregs purified from naïve PBMCs (without UCMSC-priming) of MS patients failed to reduce the proliferation of allogeneic Teffs from healthy controls while Tregs purified from UCMSC-primed PBMCs of the same MS significantly reduced the proliferation index of allogeneic Teffs from healthy donors. No significant difference in the proliferation index of allogeneic Teffs was detected in Tregs purified from UCMSC-modulated PBMCs of MS and Tregs purified from naïve PBMCs of HC, suggesting that UC-MSC-priming may have reversed the functional deficits of Tregs from MS in the co-culture. Tregs:Teffs =1:10. **p<0.01, *p<0.05. **B**, **C**, & **D**. Representative ModFit results of CFSE-labeled and PHA-stimulated allogeneic CD4+CD25- Teffs of healthy donors in co-cultures with (B) Tregs sorted from naïve PBMCs of healthy donors, (C) Tregs sorted from naïve PBMCs of MS patients, and (D) Tregs sorted from UC-MSC-primed PBMCs of the same MS patients at a constant ratio of 1:10 for Tregs:Teffs. **E**. Bar graph showing that Tregs purified from UC-MSC-primed PBMCs of MS patients significantly reduced the proliferation index of autologous Teffs compared to Tregs purified from naïve PBMCs of the same MS patients. **p<0.01. **F** & **G**. Representative ModFit results of CFSE-labeled and PHA-stimulated autologous CD4^+^CD25^−^ Teffs in co-cultures with (F) Tregs purified from naïve PBMCs of MS patients and (G) Tregs purified from UC-MSC-primed PBMCs of the same MS patients at a ratio of 1:10 for Tregs:Teffs

We evaluated the suppressive ability of CD4+CD25+CD127low/− Tregs from MS to the proliferation of CD4+CD25- Teffs stimulated by PHA (10μg/ml). Tregs from MS failed to inhibit the proliferation of PHA-stimulated allogeneic Teffs (Figure 2A, p<0.01) from heathy donors and autologous Teffs from the same MS patients (Figure 2E, p<0.05). These results demonstrated that suppressive functions are severely impaired in Tregs from MS. To investigate if UC-MSCs improve the impaired function of Tregs from MS, we co-cultured UC-MSCs with PBMCs from MS patients at a ratio of 1:5 for 3 days and then isolated CD4+CD25+CD127low/− Tregs for functional examination. UC-MSC-primed CD4+CD25+CD127low/− Tregs from MS significantly inhibited the proliferation of allogeneic CD4+CD25- Teffs from heathy donors (Figure 2A) and autologous CD4+CD25- Teffs from the same MS patients (Figure 2E). In fact, UCMSC-primed Tregs from MS reduced the proliferation index of PHA-stimulated allogeneic CD4+CD25- Teffs from heathy donors to the same degree as that produced by Tregs from heathy donors (Figure 2A); i.e., no significant difference was detected in the suppressive capacity of naïve Tregs from healthy donors and UCMSC-primed Tregs from MS patients, suggesting that UCMSC-co-cultures may have reversed the impaired suppressive function of Tregs from MS.

### UC-MSCs significantly improved regulatory cytokine production in co-cultures

**Figure 3:**
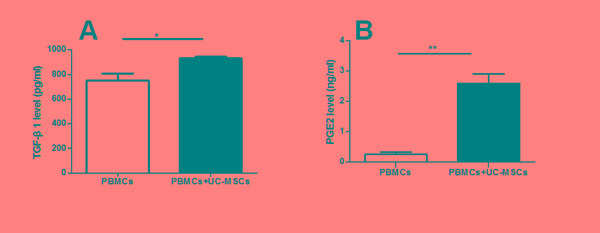
UC-MSCs significantly improved the production of cytokines in the co-cultures Bar graph showing that UC-MSCs significantly augmented the production of TGF-β1 (**A**), PGE2 (**B**), and IL-10 (**C**) while reducing the production of IFN-γ (**D**) in co-cultures of UC-MSCs and PBMCs from MS patients. Each experiment was performed in triplicate. Data were presented as mean ± SD of four independent experiments. *p<0.05, **p<0.01, ***p<0.0001.

As shown in Figure 3, UCMSC co-cultures decreased the production of pro-inflammatory cytokine IFN-γ (Figure 3D) but increased the production of the anti-inflammatory cytokines TGF-β1, PGE2, and IL-10 (Figure 3A-3C) in PBMCs from MS. In fact, IL-10 was hardly detected in the supernatant of PBMC cultures from MS patients. However, co-cultures with UC-MSCs did not result in significant differences in the production of IL-10 in PBMC samples from healthy donors (data not shown).

### UC-MSCs-priming enhanced the capacity of Tregs from MS to release IL-10

**Figure 4:**
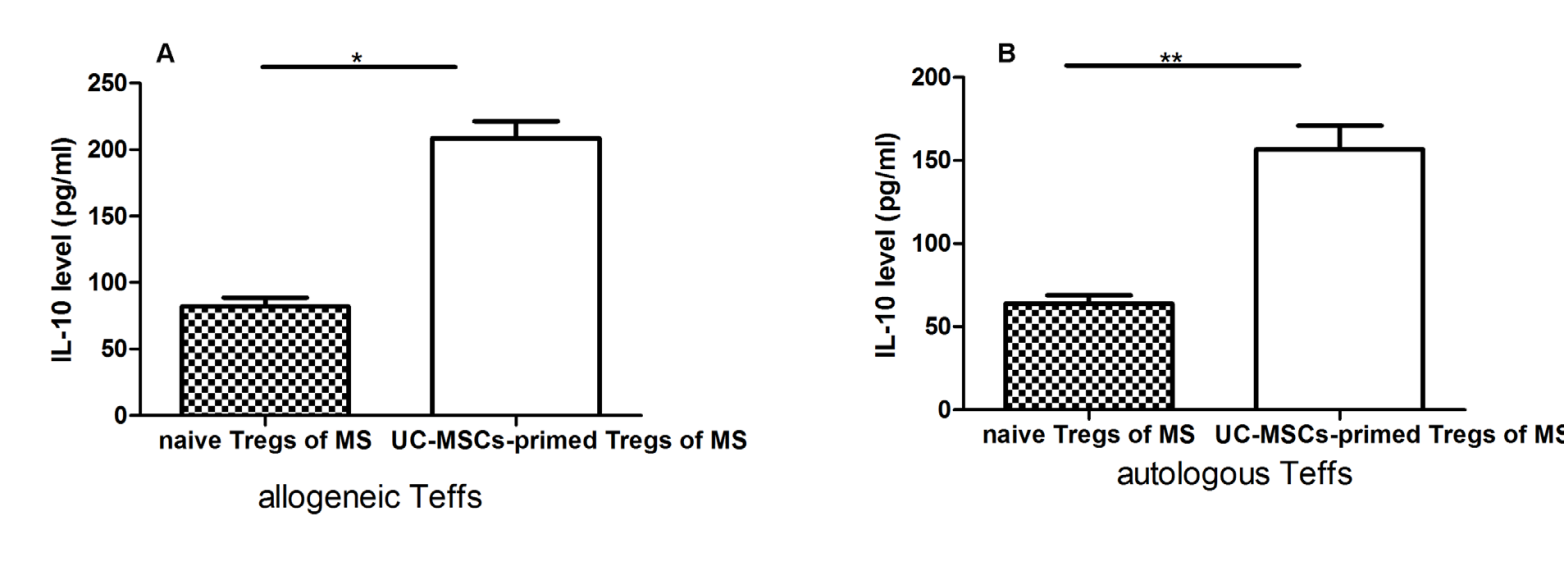
UC-MSC-primed Tregs from MS patients significantly increased IL-10 secretion levels in co-cultures with allogeneic Teffs from healthy donors or autologous Teffs from MS patients **A** & **B**: Bar graph showing that UC-MSC-primed Tregs from MS patients significantly increased IL-10 secretion levels in co-cultures with allogeneic Teffs (A) from healthy donors or autologous Teffs (B) from MS patients compared with naïve Tregs (without UC-MSC priming) from the same MS patients. Each experiment was performed in triplicate. Data were presented as mean ± SD of four independent experiments. *p<0.05, **p<0.01.

We further investigated the possible mechanism(s) of the increased suppressive effect of Tregs from UC-MSC-primed PBMCs of MS and measured IL-10 levels in the supernatant of Treg and Teff co-cultures by ELISA assay. IL-10 levels in the co-culture of Tregs from UC-MSC-modulated PBMCs of MS patients and Teffs from either autologous or allogeneic origins were significantly increased compared to the co-cultures of Tregs from naïve PBMCs of MS without considering the source origins of Teffs (Figure 4). The improved capacity of Tregs for IL-10 release may have contributed to the increased suppressive function of Tregs from MS.

## DISCUSSION

Tregs play a crucial role in homeostasis and self-tolerance control by dynamically suppressing the activation and expansion of autoreactive T-cells and other immune cells in peripheral and CNS systems [23-25]. The therapeutic potential of Tregs has been well documented in MS animal models [26, 27]. Clinical trials have demonstrated that systemic infusion of autologous Tregs in the treatment of autoimmune diseases such as type I diabetes in children is well tolerated without major side-effects [28]. However, a number of studies including our own identified multiple defects of Tregs in the peripheral blood of patients with MS and other autoimmune diseases; i.e. altered composition, reduced suppressive function, and poor secretion of inflammation-inhibiting cytokines [4, 12, 30-33]. Although no difference was observed between the frequency of Tregs from MS patients and from healthy donors in our own studies (Figure 1D), our data revealed that Tregs from MS failed to suppress the proliferation of Teffs stimulated by *in vitro* PHA (Figure 2A & 2E). That is, autologous Treg from MS as a cell therapy is unlikely to be efficacious without a correction of the intrinsic impairments in Treg function.

MSCs are a subset of multipotent stem cells capable of self-renewing and differentiating into multiple mesenchymal cell lineages for connective tissues [29-31], showing great potential in regenerative medicine. In addition, MSCs displayed multiple regulatory roles in the immune system by inducing the generation of Tregs via cell-cell contact between MSCs and T cells and secreting anti-inflammatory factors *in vitro* [20, 32], while *in vivo* studies showed that MSCs are able to control the progress of autoimmune diseases, including MS by inducing the expansion of Tregs [33]. UC-MSCs are isolated from discarded human umbilical cords, which offer an abundant and noninvasive source of MSCs without the concern of controversial ethical issues. UC-MSCs are viable alternatives to BM-derived MSCs. It has been reported that UC-MSCs share characteristics of BM-derived MSCs but are more primitive in nature with more powerful immune-modulating properties. For example, UC-MSC-suppressed mitogen induced lympho-proliferation to a greater extent than BM-derived MSCs. Furthermore, the extent of immune-suppression in inflammation is higher in UC-MSCs than in BM-derived MSCs [34]. In addition, UC-MSCs are readily available, replicate rapidly in culture with a regular doubling time, and have low levels of senescence after repeated passages [18, 19].

To repair the defects of Tregs from MS patients, we co-cultured Tregs from MS patients with UC-MSCs and found UC-MSCs not only increased the frequency of Tregs of MS (Figure 1D) but also reversed their impaired suppressive function (Figure 2A). No significant difference in the suppressive function of Tregs was detected between Tregs from UC-MSC- primed PBMCs of MS patients and Tregs from naïve PBMCs from healthy donors, suggesting that the impaired suppressive function of Tregs may have reversed.

Some scientists suggest that Teffs resistance to the regulation of Tregs may also contribute to the pathogenesis of MS [35]. We did not examine whether there is a resistance of Teffs to Tregs in our current studies. However, our data showed that UC-MSC-primed Tregs from MS patients efficiently suppressed the proliferation of autologous Teffs stimulated by PHA compared to non-UC-MSC-primed Tregs from the same MS patients (Figure 2E).

As shown in Figure 3A-3C, we found PGE2, TGF-β1, and IL-10 levels in the co-cultures of PBMCs with UC-MSCs were significantly higher than those in the cultures of PMBCs without UC-MSCs. In contrast, the levels of IFN-γ in PMBC co-cultures with UC-MSCs were significantly lower than those of PBMC cultures without UC-MSCs (Figure 3D). Thus, soluble cytokines such as PGE2, TGF-β1, IL-10, and IFN-γ contribute to the phenotypic changes of mitogen-activated lymphocytes [36]. It is possible that UC-MSCs modulate the production of cytokines primarily through cell-cell contact because the conditioned medium of UC-MSCs only partially results in cytokine production alterations (data not shown). As suggested by other investigators [37], IL-10 may be primarily responsible for the enhanced suppressive capability of MSC-exposed Tregs. Interestingly, we found that the levels of IL-10 in co-cultures of Tregs from UC-MSC-primed PBMCs of MS patients with allogenic or autologous Teffs were significantly higher than those in the co-cultures of Tregs from naïve PBMCs of the same MS patients with allogenic or autologous Teffs (Figure 4A & 4B), suggesting that the improved cytokine production capacity may have contributed to the increased frequency and suppressive function of Tregs from MS. However, we did not verify which cell population secreted IL-10 into co-cultures because of blood sample limitations. Further studies are warranted to clarify this question.

In conclusion, we report for the first time that the intrinsic Treg defect in MS can be repaired *in vitro* using a UC-MSC-mediated immune modulation. UCMSC-primed Tregs from MS can effectively inhibit the proliferation of PHA-stimulated autologous Teffs from the same MS patients and allogeneic Teffs from healthy donors with a degree of inhibition observed in Tregs from healthy donors. Our studies provide valuable preliminary *in vitro* data to support the development of functionally normalized Tregs of autologous origins from individual patients with autoimmune diseases using a simple UC-MSC-based priming approach and may offer new therapeutic treatments for MS and other autoimmune diseases.

## MATERIALS AND METHODS

### Isolation and culture of UC-MSCs

Human umbilical cords (n=3) were purchased from StemExpress (Placerville, CA, USA). We isolated and purified UC-MSCs according to the protocol previously published by our group [22]. In brief, the vessels of UC were removed to retain Wharton's jelly. Wharton's jelly tissue was then cut into 1 mm^3^ pieces, added to the bottom of the tissue culture dish (BD, Franklin Lakes, New Jersey), and incubated at 37°C in a 5% carbon dioxide incubator without culture medium for two hours to allow tissue attachment. 15ml complete culture medium containing DMEM (low glucose) supplemented with 10% FBS (fetal bovine serum), 1% L-glutamine, and 1% penicillin-streptomycin (all from Invitrogen, Grand Island, NY) was added to the tissue culture dishes and incubated for an additional 7 days at 37°C and 5% carbon dioxide incubator. The purified Wharton's jelly pieces were removed and the primary cells that had migrated out of the Wharton's jelly tissues and attached to tissue culture dishes were passaged by a 1-min treatment with 0.25% trypsin and 0.02% EDTA at 37°C. The medium was changed every 3 days. UC-MSCs were further passaged when they reached 90 confluency. All UC-MSCs used in experiments were passages 3-5.

### Co-cultures of UC-MSCs and PBMCs

PBMCs obtained from patients with relapsing-remitting multiple sclerosis (RRMS, n=12, female=8, male=4), mean age 53.75 years old (49 years to 64 years old) and healthy donors (n=10, female=4, male=6), mean age 28.38 years old (22 years to 39 years old) were purchased from AllCells Inc (Emeryville, CA) or StemExpress (Placerville, CA). All patients were under treatment; one with Copaxone, one with Tysabri, one with Avonex, and one with HCTZ. The others were under treatment with unknown drugs. The duration of the disease and prior treatment history were unknown. Before co-culture, allogenic UC-MSCs (2×10^5^) were seeded into each well of a 6-well tissue culture plate (BD), cultured in 2 ml of advanced RPMI 1640 culture medium (Invitrogen, Grand Island, NY, USA) supplemented with 10% FBS, 2 mM glutamine, 100 U/ml penicillin and streptomycin, and allowed to adhere at 37°C and 5% carbon dioxide incubator for 4h. PBMCs (1×10^6^) from MS patients and healthy donors were directly loaded onto the cultured UC-MSCs and co-cultured for 72h in a culture medium (2ml/well) containing advanced RPMI 1640 (Invitrogen, Grand Island, NY, USA) supplemented with 10% FBS (fetal bovine serum, Invitrogen), 1% L-glutamine, and 1% penicillin-streptomycin (Invitrogen, Grand Island, NY). PBMCs cultured alone (without UC-MSCs) served as controls. Each experiment was performed in triplicate. After 3 days co-culture, the suspended PBMCs were collected to perform flow analysis for CD4^+^CD25^+^CD127^low/−^ Tregs frequency and cell sorting for purified CD4^+^CD25^+^CD127^low/−^ Tregs and CD4^+^CD25^−^ T effectors (Teff). The cell-free supernatant was collected by centrifugation (1000 rpm x 5min) and kept frozen at −80°C until ELISA analysis.

### Flow cytometry and CD4^+^CD25^−^ Teffs and CD4^+^CD25^+^CD127^low/−^ Tregs purification

Flow cytometry analysis was performed according to our previous protocol [22]. In brief, 1×10^6^ suspended UC-MSC-primed or naïve PBMCs from MS patients or naïve PBMCs from healthy donors were washed with washing buffer (PBS containing 2% FBS) and incubated in 100μl washing buffer supplement with 10μl various fluorescent dye-conjugated specific antibodies (APC-conjugated mouse anti-human CD4, PE-conjugated mouse anti-human CD25, and PE-Cy7-conjugated mouse anti-human CD127) on ice for 30min. All antibodies were purchased from eBioscience Inc (USA). After incubation, 2×10^5^ suspended naïve or UC-MSC-primed PBMCs from MS patients or naïve PBMCs from healthy donors were washed twice with washing buffer, resuspended in 200μl washing buffer, and analyzed by the LSRFortessa^TM^ cell analyzer (BD, Franklin Lakes, New Jersey). The left 8×10^5^ suspended naïve or UC-MSC-primed PBMCs from MS patients or naïve PBMCs from healthy donors were used to purify CD4^+^CD25^+^CD127^low/−^ Tregs and CD4^+^CD25^−^ T effectors (Teff). Purification of CD4^+^CD25^−^ Teffs and CD4^+^CD25^+^CD127^low/−^ Tregs was performed according to the protocol of Zhao et al with minor modifications[8]. 8×10^5^ stained UC-MSC-primed or naïve PBMCs from MS patients or naïve PBMCs from healthy donors were resuspended in 200μl buffer containing PBS, 2% BSA (bovine serum albumin), and 0.02% EDTA. Separation of CD4^+^CD25^−^ Teffs and CD4^+^CD25^+^CD127^low/−^ Tregs was conducted with a Dako-Cyomation MoFlo high speed sorter (Dako, Houston, TX).

### Cell proliferation assay

Purified CD4^+^CD25^−^ Teffs from PBMCs of healthy donors or MS patients were labeled with 10μM CFSE (5,6-carboxyfluorescein diacetate succinimidyl ester, Invitrogen) in pre-warmed PBS containing 0.1% BSA at a final concentration of 1×10^6^ cells/ml for 15min at 37°C in the dark, a protocol described by our group[22]. After incubation, CFSE-labeled CD4^+^CD25^−^ Teffs were washed twice using 5 volumes of ice-cold culture media (advanced RPMI 1640 supplemented with 10% FBS, 1% L-glutamine, and 1% penicillin-streptomycin). 1×10^4^ CD4^+^CD25^+^CD127^low/−^ Tregs purified from UC-MSC-primed or naïve PBMCs of MS patients or healthy donors were co-cultured with 1×10^5^ CFSE-labeled CD4^+^CD25^−^ Teffs (Tregs:Teffs = 1:10 in 0.2ml) in the presence of PHA (10μg/ml). Five groups were included in this experiment: Tregs from naïve PBMCs of healthy donors + Teffs from naïve PBMCs of healthy donors; Tregs from naïve PBMCs of MS patients + Teffs from naïve PBMCs of healthy donors; Tregs from UC-MSC-primed PBMCs of MS patients + Teffs from naïve PBMCs of healthy donors; Tregs from naïve PBMCs of MS patients + Teffs from naïve PBMCs of MS patients; Tregs from UC-MSC-primed PBMCs of MS patients + Teffs from naïve PBMCs of MS patients. Each experiment was performed in triplicate. Three days later, cells were harvested for flow cytometry by LSRFortessa™ cell analyzer (BD, Franklin Lakes, New Jersey) at 488nm excitation. The proliferation index was calculated using ModFit Software (Verity Software House). Cell-free supernatant was collected by centrifugation (1000 rpm x 5min) and kept frozen at −80°C until ELISA analysis.

### Cytokine analysis by ELISA

Cell-free supernatant was collected after co-culture of PBMCs with UC-MSCs (1:5) and cultures of PBMCs without UC-MSCs for 3 days and used to measure the concentrations of cytokine prostaglandin E2 (PGE2), transforming growth factor (TGF)-β1, interleukin−10 (IL−10), and interferon (IFN)-γ by ELISA analysis according to the manufacturer's protocol. Cell-free supernatant collected after Tregs and Teffs (1:10) co-cultures for 3 days were used to measure the concentration of cytokine IL−10 by ELISA analysis according to the manufacturer's protocol. TGF-β1, IL−10, and IFN-γ ELISA kits were purchased from eBioscience (San Diego, CA), and the PGE2 ELISA kit was purchased from R&D System (Minneapolis, CA). The collected cell-free supernatant was placed in a 96-well specific antibody-coated plate. Absorbance was recorded at 450 nm using a microplate reader (Bio-Rad, Philadelphia, PA). Each cytokine standard and sample was run in duplicate.

### Statistical analysis

Statistical analysis was performed using GraphPad Prism 5 (GraphPad). Data were expressed as means ±SEM. A two-sample T test or one-way ANOVA was used for statistical analysis. A p-value <0.05 was considered significant.
